# Mitochondrial Disorder Aggravated by Metoprolol

**DOI:** 10.1155/2016/7869174

**Published:** 2016-10-20

**Authors:** Cheryl Samuels, Mary Kay Koenig, Mariana Hernandez, Aravind Yadav, Ricardo A. Mosquera

**Affiliations:** Department of Pediatrics, University of Texas Medical School at Houston, Houston, TX, USA

## Abstract

Beta-adrenergic blocking agents or beta-blockers are a class of medications used to treat cardiac arrhythmias and systemic hypertension. In therapeutic dosages, they have known adverse outcomes that can include muscular fatigue and cramping, dizziness, and dyspnea. In patients with mitochondrial disease, these effects can be amplified. Previous case reports have been published in the adult population; however, their impact in pediatric patients has not been reported. We describe a pediatric patient with a mitochondrial disorder who developed respiratory distress after metoprolol was prescribed for hypertension. As the patient improved with discontinuation of medication and no alternative etiology was found for symptoms, we surmise that administration of metoprolol aggravated his mitochondrial dysfunction, thus worsening underlying chest wall weakness.

## 1. Introduction

Mitochondrial disorders are a group of diseases characterized by dysfunction of the mitochondria, the organelles responsible for generating the energy required to sustain life and support growth [1]. Mitochondria are present throughout our body and their dysfunction can affect any organ, including the brain, heart, skeletal muscle, or respiratory system; symptoms may include loss of motor control, muscle weakness, failure to thrive, cardiac disease, gastrointestinal disorders, and seizures, among others. Diagnosis of a mitochondrial disease is made via a combination of clinical, laboratory, and molecular analysis and often involves the use of standardized diagnostic criteria [2, 3]. There is no specific therapy for mitochondrial disease. Treatment focuses on nutritional supplements that theoretically improve mitochondrial function along with optimization of care during times of stress and avoidance of substances that are known to be toxic to the mitochondria themselves [4]. A recent guideline released by the Mitochondria Medical Society helps to standardize evaluation, diagnosis, and care of these patients [5].

Beta-adrenergic blocking agents or beta-blockers are a class of medications used to treat cardiac arrhythmias and systemic hypertension. Beta-blockers' pharmaceutical action lies with their ability to block the effects of epinephrine. This blockage causes the heart to beat slower and less forcefully, thereby decreasing blood pressure. Known adverse outcomes include fatigue, dizziness, dyspnea, and bradycardia. Beta-blockers have a strong potential to damage mitochondrial biogenesis. The damage is assumed to be due to direct effect on the respiratory electron transport chain with decreased levels of arginine impairing aerobic exercise capacity [6, 7]. These medications can also inhibit the biological pathway of coenzyme Q10 (CoQ10). CoQ10 is a mitochondrial coenzyme essential for the production of adenosine triphosphate (ATP), the basis of cellular energy processes. CoQ10 also scavenges free radicals and blockage of its action interferes with protection against free radicals, contributing to further mitochondrial dysfunction [8].

Skeletal muscle predominantly expresses *β*_2_-receptors and the use of a *β*-blocker limits aerobic muscle pathways by decreasing oxygen availability to the muscle. Clinically, these drugs have been associated with muscle cramps, muscular weakness, generalized fatigue, myopathy, lactic acidosis, and elevation of creatinine kinase levels [9]. Included in the potentially affected muscles are the diaphragm and chest wall muscles where weakness can negatively impact respiratory status.

## 2. Case Presentation

We present the case of a 7-year-old Caucasian boy diagnosed clinically with a mitochondrial disorder. The patient presented with multisystem disease including autonomic dysfunction, gastroesophageal reflux, gastroparesis, nocturnal hypoventilation, and metabolic derangements including elevations in lactate and pyruvate. Muscle biopsy found a global reduction (<25%) in all enzyme complexes of the electron transport chain and the patient was placed in a category of* definite* mitochondrial disease using the modified Walker criteria [2, 3].

At his baseline, the patient had a sleep related breathing disorder with hypoventilation, characterized by hypercapnia with end tidal carbon dioxide levels (EtCO_2_) of more than 50 torr for greater than 25% of the measured sleep time. A nocturnal polysomnography study performed immediately prior to the acute decompensation period revealed continued hypoventilation with hypoxemia (average oxygen levels were 88% with a nadir of 73% without breathing support) and hypercapnia (EtCO_2_: 49–65) with an average of 3 central apneas per hour. In order to treat the sleep disturbances noninvasive ventilation was initiated with Bilevel Positive Airway Pressure (BiPap) at settings of 12/6 cmH_2_O.

Prior to his acute respiratory decompensation he was doing well, at his medical baseline, was active, and was attending school. His mother reported headaches associated with elevated blood pressure (averaging 135/88: stage 2 hypertension) a reason why metoprolol was prescribed at a dose of 25 mg (1 mg/kg) by his pediatric cardiologist for treatment of both hypertension and dysautonomia.

After the treatment with metoprolol was initiated, blood pressure returned to normal levels. Within weeks the child's mother reported intermittent, mild episodes of dyspnea exacerbated by activity and associated with periodic grunting and generalized weakness. These symptoms persisted and worsened and, two months following the initiation of metoprolol, he was admitted to the pediatric intensive care unit with acute respiratory insufficiency.

The child's mother reported that on the day of admission his breathing became rapid and shallow and the presence of a thoracoabdominal asynchronous breathing pattern was noted. At the time of admission, he was alert, active, and cooperative, with his baseline activity and oral intake. He was not experiencing any evidence of infection: no fever, cough, rhinorrhea, or sputum production. Later his breathing becomes more labored and erratic with a respiratory rate of 7-8 breaths per minute. On physical examination no wheezing or crackles were auscultated. His oxygen saturations remained above 90%, and his EtCO_2_ levels were 34 mmHg. Difficulty breathing and thoracoabdominal asynchrony were evident. Oxygen therapy was initiated and BiPap settings were increased and expanded to 24-hour coverage.

Studies performed were normal. Specifically, a CBC gave no evidence of infection. Blood gases at time of PICU admission revealed a pH of 7.44, pCO_2_ 34.8. A chest X-ray and a CT scan of the lung revealed normal lung parenchyma. EEG showed no abnormalities and CT of the brain was normal. He was placed on an arterial line for continuous monitoring of his arterial blood gases, which remained within normal limits and his lactic acid level was at 1.7 mmol/L.

He was discharged after 4 days and sent home with BiPap with settings of 14/5 cm H_2_O for use while asleep and when needed while awake with supplemental oxygen therapy. He continued to experience mild episodes of hypoventilation and asynchronous breathing a week following discharge and was evaluated by his pediatric pulmonologist.

At this point, no clear origin for his respiratory symptoms had been found. Specifically, he continued with the thoracoabdominal asynchrony accompanied by the absence of abnormal chest sounds with normal chest CT, chest X-ray, and normal carbon dioxide and oxygen levels. Negative findings suggested a chest wall weakness disorder rather than lung parenchymal disease. In order to exclude a possible relationship between the beta-blockers and the chest wall weakness, metoprolol was discontinued. As an alternative diagnosis to the chest wall weakness was central dysventilation was considered less likely based on the recent sleep study without evidence of central sleep apnea.

After discontinuation of the metoprolol, dyspnea and abdominal breathing pattern gradually improved. He returned to his medical baseline within four weeks after discontinuation of the medication.

## 3. Discussion

In the present case, our pediatric mitochondrial patient developed respiratory insufficiency in the presence of reassuring lab values. In children with respiratory failure associated with neuromuscular disease, the initial arterial carbon dioxide tension can drop to levels at or below the normal range. This phenomenon reflects alveolar hyperventilation which is most likely produced by a combination of fear, small airway collapse, and reflex tachypnea. It is only later in the development of severe respiratory weakness that alveolar ventilation fails and carbon dioxide tension rises to first normal and then elevated levels. In our patient noninvasive mechanical ventilation was started in the early stages of respiratory failure while his pCO_2_ was still within the normal range (34 mmHg).

Review of the medical literature found a previous case report of an adult mitochondrial patient who developed muscular weakness, generalized fatigue, and muscle cramps after starting a beta-blocker [10]. An additional report identified beta-blockers as mitochondrial-toxic agents, causing a significant impact in patients with mitochondrial disorders [11]. With no other cause apparent to explain our patient's respiratory decompensation, the decision was made to discontinue the medication and symptoms resolved.

Based on these previous reports and our clinical scenario, we speculate that beta-blockers can aggravate or trigger muscle weakness in patients with a mitochondrial disease. Given the absence of studies and case reports regarding the use of beta-blockers in pediatric mitochondrial patients, the main objective of this report is to alert providers that beta-blockers should be used with caution in patients with mitochondrial disease as these agents may trigger or further aggravate muscle manifestations.

## Abbreviations

CoQ10: Coenzyme Q10
ATP: Adenosine triphosphate
Β: Beta
BiPap: Bilevel Positive Airway Pressure
EtCO_2_: End tidal carbon dioxide.
